# Direct antiviral properties of TLR ligands against HBV replication in immune-competent hepatocytes

**DOI:** 10.1038/s41598-018-23525-w

**Published:** 2018-03-29

**Authors:** Julie Lucifora, Marc Bonnin, Ludovic Aillot, Floriane Fusil, Sarah Maadadi, Laura Dimier, Maud Michelet, Océane Floriot, Anaïs Ollivier, Michel Rivoire, Malika Ait-Goughoulte, Stéphane Daffis, Simon P. Fletcher, Anna Salvetti, François-Loïc Cosset, Fabien Zoulim, David Durantel

**Affiliations:** 1 INSERM, U1052, Cancer Research Center of Lyon (CRCL), Université de Lyon (UCBL1), CNRS UMR_5286, Centre Léon Bérard, Lyon, France; 2 CIRI – International Center for Infectiology Research, Inserm, U1111, Université Claude Bernard Lyon 1, CNRS, UMR5308, Ecole Normale Supérieure de Lyon, Univ Lyon, F-69007 Lyon, France; 3 INSERM U1032, Centre Léon Bérard (CLB), Lyon, France; 4 Roche Pharma Research and Early Development (pRED), Roche Innovation Center Basel, F. Hoffmann-La Roche, 4070 Basel, Switzerland; 5 Gilead Sciences, Inc, Foster City, CA USA; 6 Department of Hepatology, Croix-Rousse Hospital, Hospices Civils de Lyon, Lyon, France

## Abstract

Current therapies for chronic hepatitis B virus (HBV) infections are effective at decreasing the viral load in serum, but do not lead to viral eradication. Recent studies highlighted the therapeutic or “adjuvant” potential of immune-modulators. Our aim was to explore the direct anti-HBV effect of Toll-Like-Receptors (TLR) agonists in hepatocytes. HBV-infected primary human hepatocytes (PHH) or differentiated HepaRG cells (dHepaRG) were treated with various TLR agonists. Amongst all TLR ligands tested, Pam3CSK4 (TLR1/2-ligand) and poly(I:C)-(HMW) (TLR3/MDA5-ligand) were the best at reducing all HBV parameters. No or little viral rebound was observed after treatment arrest, implying a long-lasting effect on cccDNA. We also tested Riboxxol that features improved TLR3 specificity compared to poly(I:C)-(HMW). This agonist demonstrated a potent antiviral effect in HBV-infected PHH. Whereas, poly(I:C)-(HMW) and Pam3CSK4 mainly induced the expression of classical genes from the interferon or NF-κB pathway respectively, Riboxxol had a mixed phenotype. Moreover, TLR2 and TLR3 ligands can activate hepatocytes and immune cells, as demonstrated by antiviral cytokines produced by stimulated hepatocytes and peripheral blood mononuclear cells. In conclusion, our data highlight the potential of innate immunity activation in the direct control of HBV replication in hepatocytes, and support the development of TLR-based antiviral strategies.

## Introduction

Hepatitis B virus (HBV) is a small DNA virus that persists within hepatocytes thanks to the establishment and maintenance of a covalently-closed-circular DNA (cccDNA). This cccDNA serve as the main template for viral RNA synthesis, including the pre-genomic RNA (pgRNA), which is subsequently converted into relaxed-circular DNA (rcDNA) by a HBV polymerase-mediated reverse-transcription step taking place inside nucleocapsids. Different viral particles and antigens circulate in the blood of infected patients, including HBe antigens (HBeAg), Dane particles (infectious particles or virions), spheres and rod (empty enveloped particles); the later three features envelope proteins at their surface and all comprise the pool of secreted HBs antigens (HBsAg). Nucleic acid-free subviral particles are produced in large excess compared to virions, and therefore are thought to play an important role in terms of immune subversion^1^.

With around 250 million people chronically infected, who are at high risk to develop severe liver diseases, such as cirrhosis or hepatocellular carcinoma (HCC), HBV infection remain a major medical burden worldwide. Current therapies for chronic HBV infections, mainly relying on nucleos(t)ides analogues (e.g. entecavir, tenofovir…), are effective at suppressing viremia in blood of patients and somehow improve long-term outcome, but have only low rates of HBsAg loss, with or without associated anti-HBs seroconversion, and typically do not lead to cccDNA elimination^2^. Moreover, there are life-long treatments, as drug administration arrest almost universally leads to a rebound in viremia and liver diseases. This warrants the identification of new antiviral strategies, including immune-therapeutic components, to improve the *functional cure* rates and decrease further the risk of end-stage liver diseases.

Infection of human cells by microorganisms initially leads to the activation of the host innate immune response through a sensing mediated by pattern recognition receptors (PRR). PRR include Toll-like receptors (TLR), C-type lectin receptors (CLR), RIG-I-like receptors (RLR) and NOD-like receptors (NLR), intracellular DNA sensors and cytoplasmic RNA helicases such as RIG-I (Retinoic-acid Inducible Gene-I) and MDA-5 (melanoma differentiation-associated gene-5)^3^. Each PRR detects pathogen-associated molecular patterns (PAMP) derived from viruses, bacteria, mycobacteria, fungi or parasites and activates downstream signaling events leading to specific gene expression programs and the secretion of interferons (IFN), inflammatory cytokines/chemokines, and other antimicrobial peptides^3^. Many IFNs and pro-inflammatory cytokines have been shown to have direct anti-HBV effect in hepatocytes^4^. The use of PRR agonists, in particular TLR7-L (TLR7-Ligands), to induce endogenous IFNs and other cytokines, has been successfully applied to animal models of HBV infection. It is worth noting that the sub-cutaneous injection of Pegylated-IFN-α is currently a therapeutic option for treating CHB patients, although it is associated with many adverse effects^5^. The induction of endogenous IFN-α might therefore be of interest to increase the antiviral effect and lower side effects. It was shown both in HBV-infected chimpanzees and WHV-infected woodchucks that the orally delivered TLR7 agonist GS-9620 significantly reduced viremia and HBsAg^6–8^. Moreover, GS-9620 also impacted on cccDNA expression, and led to anti-HBs seroconversion in WHV-infected woodchucks^6–8^. In human, GS-9620 was shown to be safe^9^, but its anti-HBV effect was modest at used doses (AASLD 2017). TLR9 ligands are likely to be used in the HBV field as adjuvants for HBV prophylactic vaccination^10,11^, but have also been recently shown to induce a weak antiviral activity in mono-therapy in animal models of HBV infection^12,13^. However, neither TLR7 nor TLR9 are expressed in hepatocytes^14^ suggesting that their antiviral effect results from the activation of non-hepatocyte cells, such as plasmacytoid dendritic cells, and the endogenous action of IFN and other cytokines. We previously showed that hepatocytes (primary human hepatocytes (PHH) and differentiated HepaRG cells (dHepaRG)) express a number of other PRR and that their agonization could inhibit HBV replication when the ligands were applied before viral infection and maintained throughout the experiment^14^. To provide further insight into the potential of TLR agonists to induce efficient and durable antiviral effect in already established HBV infections, we tested the direct anti-HBV effect of different TLR agonists in HBV-infected hepatocytes *in vitro*, and extended the testing for TLR2-L and TLR3-L *in vivo*.

## Results

### Antiviral effect of various TLR agonists *in vitro*

**Figure 1:**
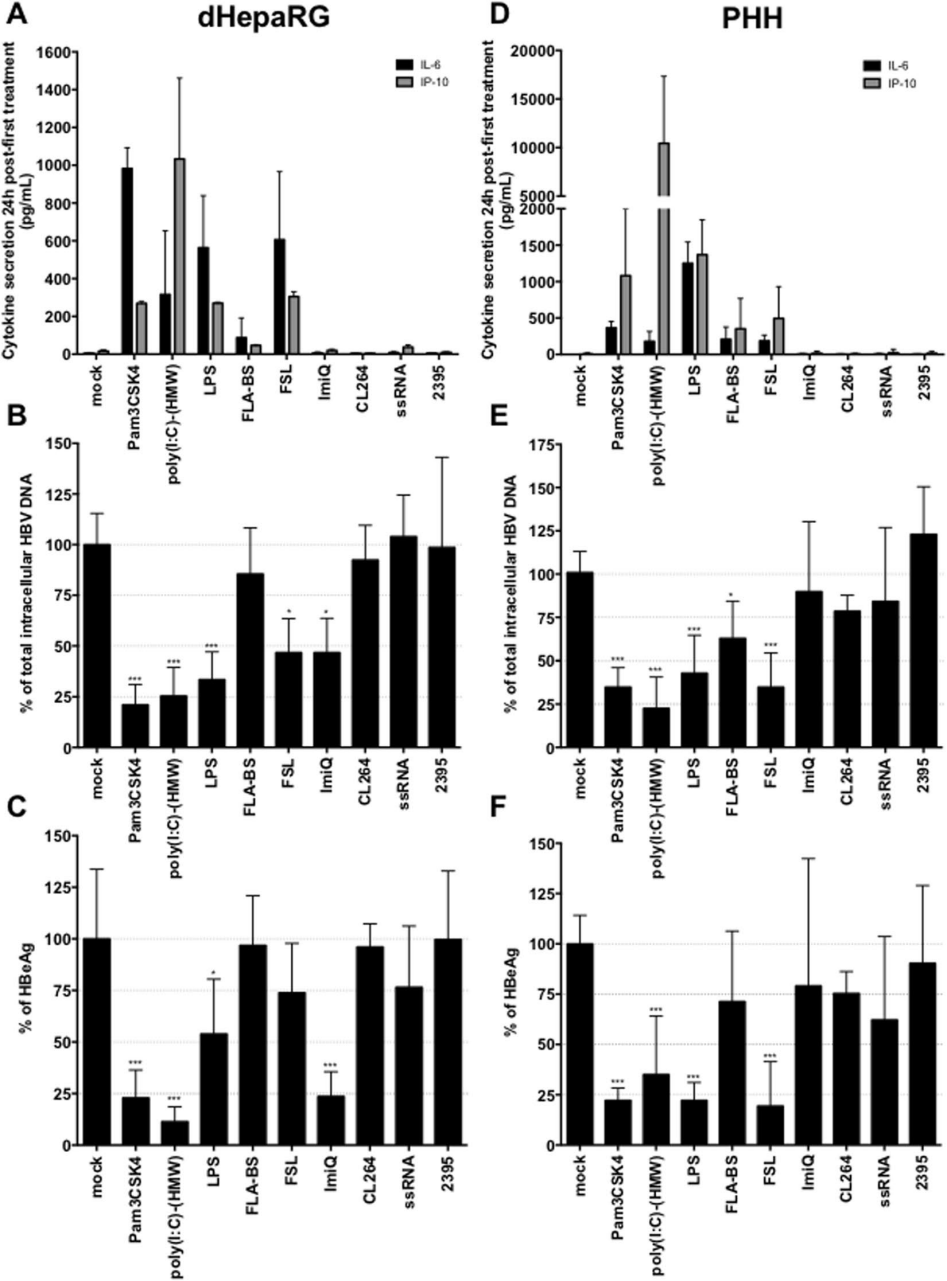
Antiviral activity of TLR-agonists in HBV-infected primary human hepatocytes (PHH) and differentiated HepaRG cells (dHepaRG). (**A**–**C**) dHepaRG or (**D**–**F**) PHH were infected by HBV at a multiplicity of infection of 100 viral genome equivalent (vge)/cell for (**A**–**C**) seven days or (D–F) 4 days, then treated twice with the indicated molecules (see method part for concentrations) for seven additional days. (**A**,**D**) Twenty-four hours after the first treatment, a fraction of cell supernatants was collected, and IL-6 and IP-10 contents were quantified by ELISA. (**B**,**E**) At the end of treatments, cells were harvested, total DNAs extracted, and total intracellular HBV DNA amounts quantified by qPCR. (**C**,**F**) Supernatants were also collected and HBeAg levels assessed by ELISA. Results are the mean +/− SD of three independent experiments (three batches of dHepaRG and three donors of PHH), each performed with three biological replicates.

To assess the effects of TLR stimulation on established HBV infection, HBV-infected PHH and dHepaRG cells were treated with Pam3CSK4 (TLR-1/2 agonist), poly(I:C)-HMW (TLR3-agonist), LPS (TLR-4 agonist), FLA-BS (TLR-5 agonist), FSL-1 (TLR-2/6-agonist), Imiquimod (ImiQ, TLR-7 agonist), CL264 (TLR-7 agonist), ssRNA (TLR-8 agonist) or 2395 (CpG ODN, TLR-9 agonist). As expected from our previous study and the pattern of TLR expression in dHepaRG and PHH^14^, cells produced significant amount of IL-6 (Figs 1A,D and S1) and IP-10 (Fig. 1A and D) mainly in response to Pam3CSK4, poly(I:C)-(HMW), LPS and FSL. Pam3CSK4, poly(I:C)-(HMW) and LPS treatments significantly decreased both total intracellular HBV DNA (Fig. 1B and E) and HBeAg secretion (Fig. 1C and F) in both HBV-infected dHepaRG cells or PHH. Treatments with FSL induced a 55–65% reduction of total intracellular HBV DNA in both HBV-infected dHepaRG and PHH (Fig. 1B), but only a significant effect on HBeAg secretion in PHH (Fig. 1C and F). As hepatocytes do not express TLR7^14^, TLR7 ligands (Imiquimod; ImiQ or CL264) induced neither IL-6 nor IP-10 following stimulation of dHepaRG. However, surprisingly, ImiQ treatment decreased total intracellular HBV DNA (Fig. 1B) as well as secretion of HBeAg (Fig. 1C) in dHepaRG, although not in PHH. This phenotype in dHepaRG was nevertheless independent of an innate response, as no production of cytokines was detected (Fig. 1A and D); we therefore did not pursue investigations on this agonist *in vitro*, as the phenotype in PHH is more relevant and likely more predictive as compared to that obtained with dHepaRG cells. As Pam3CSK4 and poly(I:C)-(HMW) were the most potent inhibitors of HBV in both *in vitro* models (Fig. 1), we further focused on the characterization of their antiviral effects for the rest of the study.

### Dose-response antiviral effect, in the absence of toxicity, of Pam3CSK4 and poly(I:C)-(HMW)

Both Pam3CSK4 and poly(I:C)-(HMW) induced a dose-dependent reduction of total intracellular HBV DNA, as well as extracellular HBeAg and HBsAg secretions without significant toxicity (Fig. S2, panels A and B). It is worth noting that, despite an absence of cytotoxicity, a significant reduction in apolipoprotein B secretion suggests that Pam3CSK4 and poly(I:C)-(HMW) could altered hepatocyte differentiation status (Fig. S2, bottom graphs of panel A). However, this phenomenon was similar to that observed with IFN-α treatment, which is used to treat CHB patients.

### Long lasting antiviral effect of Pam3CSK4 and poly(I:C)-(HMW)

**Figure 2:**
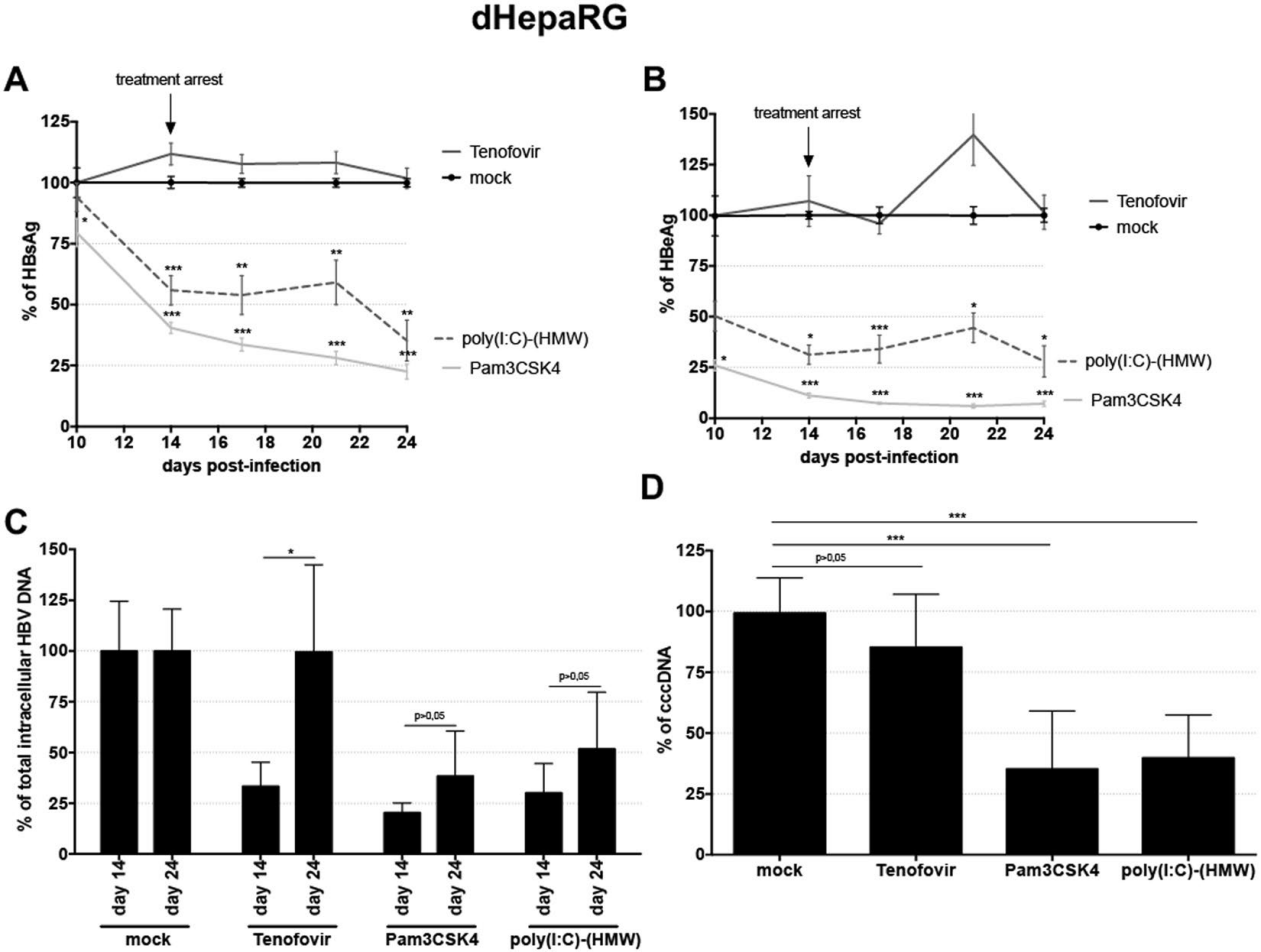
Inhibition of HBV by Pam3CSK4 and poly(I:C)-(HMW) is maintained after treatment arrest in dHepaRG cells. dHepaRG cells were infected by HBV at a multiplicity of infection of 100 viral genome equivalent (vge)/cell for seven days, treated twice with the indicated molecules for seven days, and further cultured without treatment for 10 additional days. Supernatant were collected at the indicated times and (**A**) HBsAg and (**B**) HBeAg levels assessed by ELISA. Cells were harvested at (**C**) day-14 or (**C**,**D**) day-24 post-infection, total DNAs extracted, and (**C**) total intracellular HBV DNAs or (**D**) cccDNA amounts quantified by qPCR. Results are the mean +/− SD of at least three independent experiments each performed with three biological replicates.

In contrast to the rebound observed after the arrest of nucleos(t)ides analogues^15^, reduction of total intracellular HBV DNA, HBeAg and HBsAg secretions after Pam3CSK4 or poly(I:C)-(HMW) treatment of dHepaRG cells were sustained for at least 10 days after the end of treatment (Fig. 2A–C). Additionally, using qPCR or Southern blot analyses, we observed a decrease in cccDNA levels in HBV-infected dHepaRG treated with Pam3CSK4 or poly(I:C)-(HMW) (Figs 2D and S3). Importantly, this decrease of cccDNA following Pam3CSK4 or poly(I:C)-(HMW) treatment could be confirmed in two out of three experiments using three different donors of PHH (Fig. S4A).

### Riboxxol: a novel TLR3 agonist with potent anti-HBV activity

**Figure 3:**
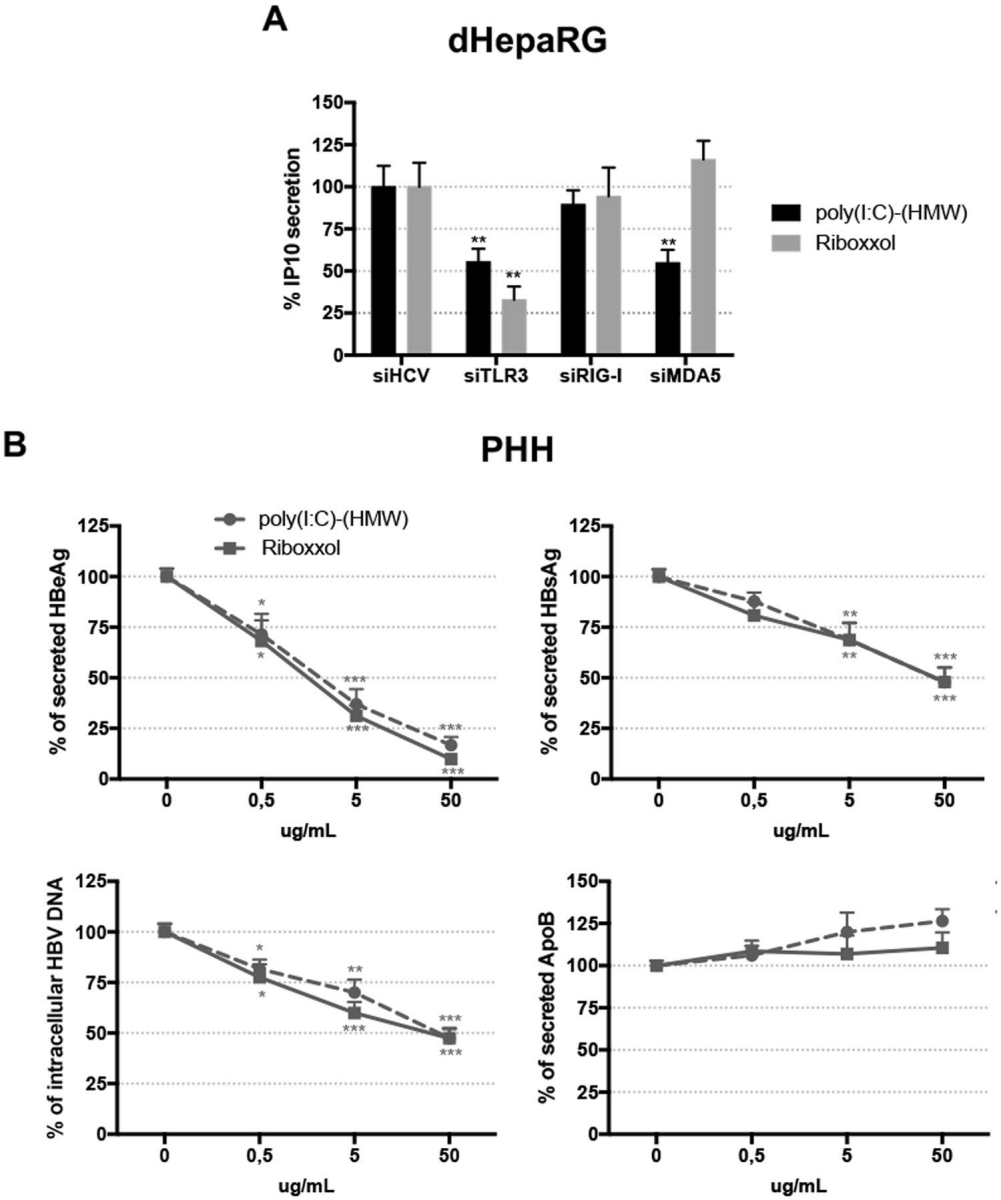
Inhibition of HBV by PolyIC(HMW) and Riboxol. (**A**) Uninfected dHepaRG cells were transfected with the indicated siRNA. 14 days later, cells were stimulated with 10 µg/ml of the indicated ligands. Cells were lyzed 24 h later and IP10 secretions were assessed by ELISA. Results are the mean +/− SD of three independent experiments each performed in technical duplicates. (**B**) PHH were infected by HBV at a multiplicity of infection of 100 viral genome equivalent (vge)/cell for seven days and treated twice with poly(I:C)-(HMW) or Riboxxol at the indicated concentration for seven days. At the end of the experiments, supernatants were collected, and HBeAg, HBsAg and ApoB levels assessed by ELISA. Cells were harvested, total DNAs extracted and total intracellular HBV DNA amounts were quantified by qPCR. Results are the mean +/− SD of four independent experiments (four donors of PHH) each performed with three biological replicates.

As poly(I:C)-(HMW) can also activate MDA5 (Fig. 3A), we tested Riboxxol, a synthetic 50 base pair double-stranded RNA, which is a specific ligand of TLR3 (Figs 3A and S5). Similarly to poly(I:C)-(HMW), treatments of HBV-infected PHH with Riboxxol dose-dependently decreased total intracellular HBV DNA, as well as extracellular HBeAg and HBsAg (Fig. 3B). Of note, a weak decrease in cccDNA levels was observed in two out of four experiments using four different donors of PHH (Fig. S4B), thus suggesting a weaker activity on this marker as compared to poly(I:C)-(HMW). Interestingly, besides its resistance to body fluids and its relevant physiochemical characteristics, which allow *in vivo* injection^16^, Riboxxol did not affect hepatocyte viability and differentiation (assessed by ApoB secretion), in contrast to Pam3CSK4 and poly(I:C)-(HMW) ligands (Figs 3B and S6).

### Kinetics of inhibition and pathways involved

**Figure 4:**
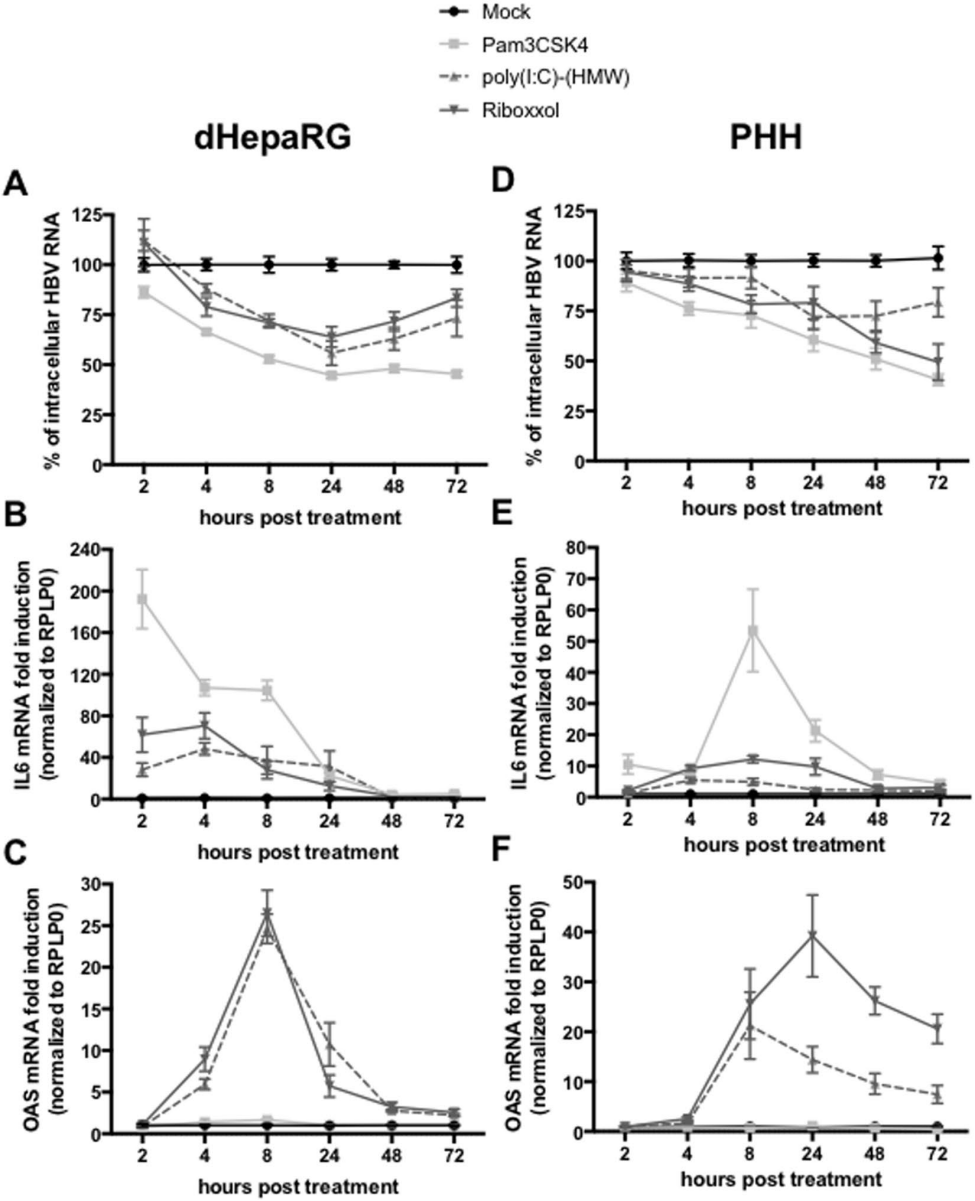
Riboxxol induced both type I IFN and NFkB pathways. (**A**–**C**) dHepaRG cells or (**D**–**F**) PHH were infected by HBV at a multiplicity of infection of 100 viral genome equivalent (vge)/cell for seven days and treated twice or not with Pam3CSK4 (1 ug/mL), poly(I:C)-(HMW) (20 ug/mL for dHepaRG, 5 ug/mL for PHH) or Riboxxol (50 ug/mL for dHepaRG, 5 ug/mL for PHH) for seven days. At the indicated time post-treatment, cells were harvested, (**A**,**D**) total RNAs extracted and levels of HBV RNA, (**B**,**E**) IL-6 mRNA or (**C**,**F**) OAS mRNA assessed by RT-qPCR. Results are the mean +/− SEM of three independent experiments (three batches of dHepaRG and three donors of PHH) each performed with three biological replicates.

The antiviral effect of Pam3CSK4 was rapid, with a 50% reduction of total HBV RNAs in HBV-infected dHepaRG or PHH (measured at 24 h or 48 h post-treatment, respectively) with a single administration of the compound (Fig. 4A,D). The reduction in HBV RNAs, following a single dose of a TLR3 ligand, was less efficient and inhibition of shorter duration in HBV-infected dHepaRG (Fig. 4A,D). As expected Pam3CSK4 treatment induced a transient increase of IL-6 mRNA, but not OAS1 mRNA levels, two prototypic genes of NF-κB and IFN pathways. In contrast, poly(I:C)-(HMW) mostly induced a transient increase of OAS mRNA level (Fig. 4B,C,E,F). Interestingly, Riboxxol induced similar (or higher) OAS mRNA level relative to poly(I:C)-(HMW), but slightly higher IL-6 mRNA level (2 fold more), as compared to poly(I:C)-(HMW) (Fig. 4B,C,E,F). This correlated well with the pattern of IL-6 and IP-10 secretion (Fig. S7). Altogether, those data indicate that Riboxxol and poly(I:C)-(HMW) can activate both NF-κB and type-I IFN pathways.

### The antiviral effect of TLR2 and TLR3 ligands in hepatocyte cultures is not due to type-I IFN and IL-6 production

**Figure 5:**
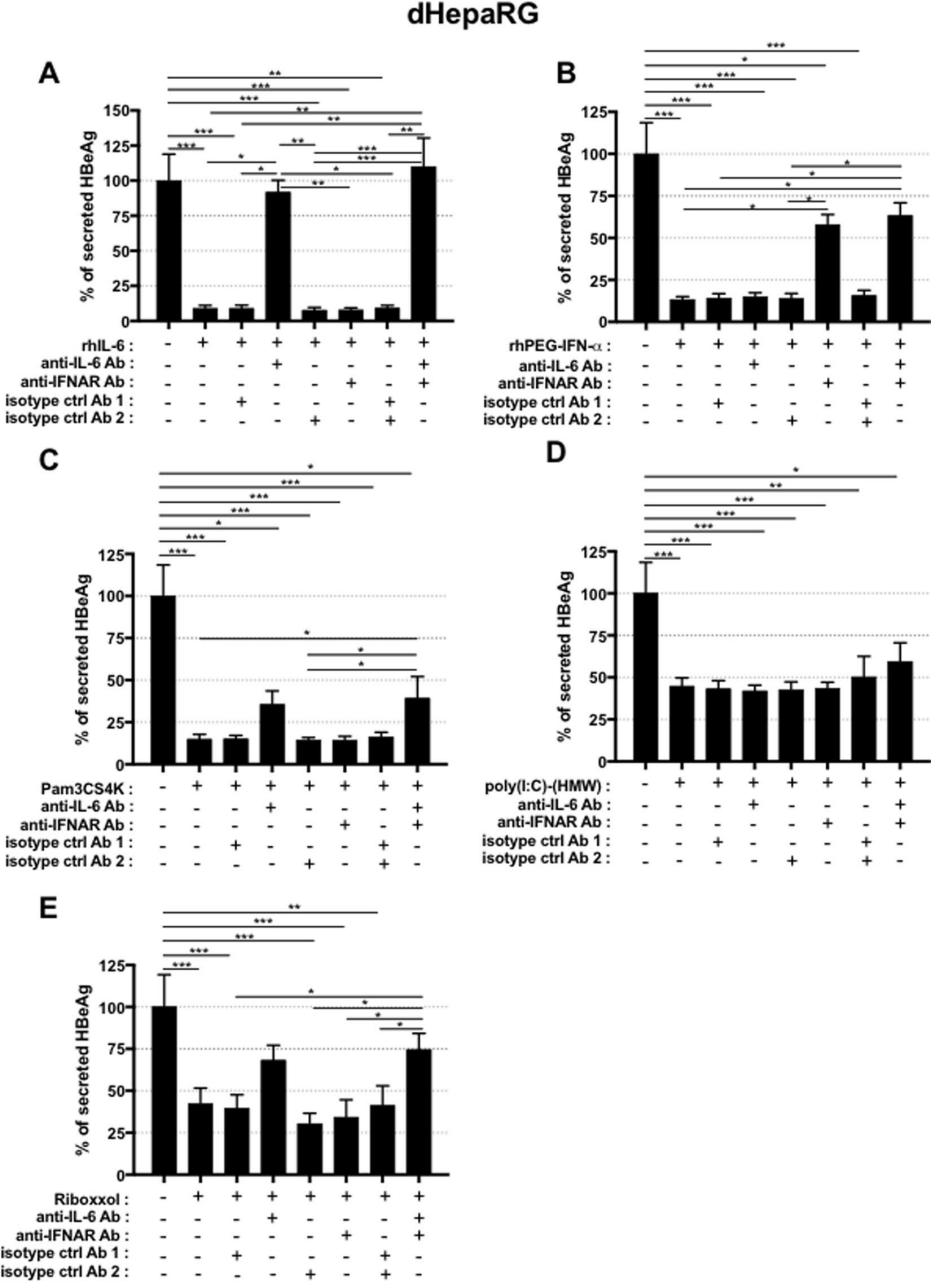
Inhibition of HBV by TLR-L is partially due to IL-6 secretion. dHepaRG cells were infected by HBV at a multiplicity of infection of 100 viral genome equivalent (vge)/cell for seven day and treated, or co-treated, twice as indicated with (**A** to **E**) anti-IL-6 antibodies (Ab) (2 ug/mL), (**A** to **E**) anti-IFNAR1 Ab (2 ug/mL), (**A** to **E**) isotype control (ctrl) Abs (1 for control of anti-IL6 Ab, 2 for control of anti-IFNRA1 Ab) (2 ug/mL), (**A**) rhIL-6 (100 ng/mL), (**B**) rhPEG-IFN-α (500 IU/mL), (**C**) Pam3CSK4 (1 ug/mL), (**D**) poly(I:C)-(HMW) (20 ug/mL) or (**E**) Riboxxol (50 ug/mL) for seven more days (2 treatments). At the end of the experiment, supernatants were collected and HBeAg levels assessed by ELISA. Results are the mean +/− SD of two independent experiments each performed with three biological replicates.

IL-6 is the main cytokine produced by hepatocytes in response to TLR1/2 or TLR3 ligands (Fig. S1). It was previously shown that IL-6 and/or type-I IFN have antiviral activity against HBV^4,15,17–19^, which was confirmed here (Fig. 5A and B). In order to investigate if secretion of IL-6 and/or type-I IFN by the stimulated hepatocytes contribute to the observed antiviral phenotypes of Pam3CSK4, poly(I:C)-(HMW) or Riboxxol, dHepaRG cells were treated with these ligands together with blocking antibodies against IL-6, IFNAR1 or both. We confirmed that the amount of anti-IL-6 antibody used was able to fully neutralize (Fig. 5A) the antiviral effect of a dose of rIL-6 hundred times higher than that secreted by dHepaRG cells (Figs 5A and S1,S7). The amount of anti-IFNAR1 antibody used was able to substantially decrease the antiviral effect of a dose of rhPEG-IFN-α at least twenty times higher than the level of type-I IFN produced by hepatocytes (Figs S1 and 5B). The anti-IFNAR1 antibody did not rescue the antiviral effect of Pam3CSK4, poly(I:C)-(HMW), or Riboxxol (Fig. 5C–E). The neutralizing anti-IL-6 antibody only induced a slight rescue of HBeAg when combined with Pam3CSK4 or Riboxxol (Fig. 5C–E). These data indicate that hepatocyte-produced IL-6 or type-I IFN are not the main drivers of the antiviral effect of Pam3CSK4, poly(I:C)-(HMW) or Riboxxol.

### Cytokines produced by immune cells upon TLR2 or TLR3 stimulation and antiviral effect in mice models

**Figure 6:**
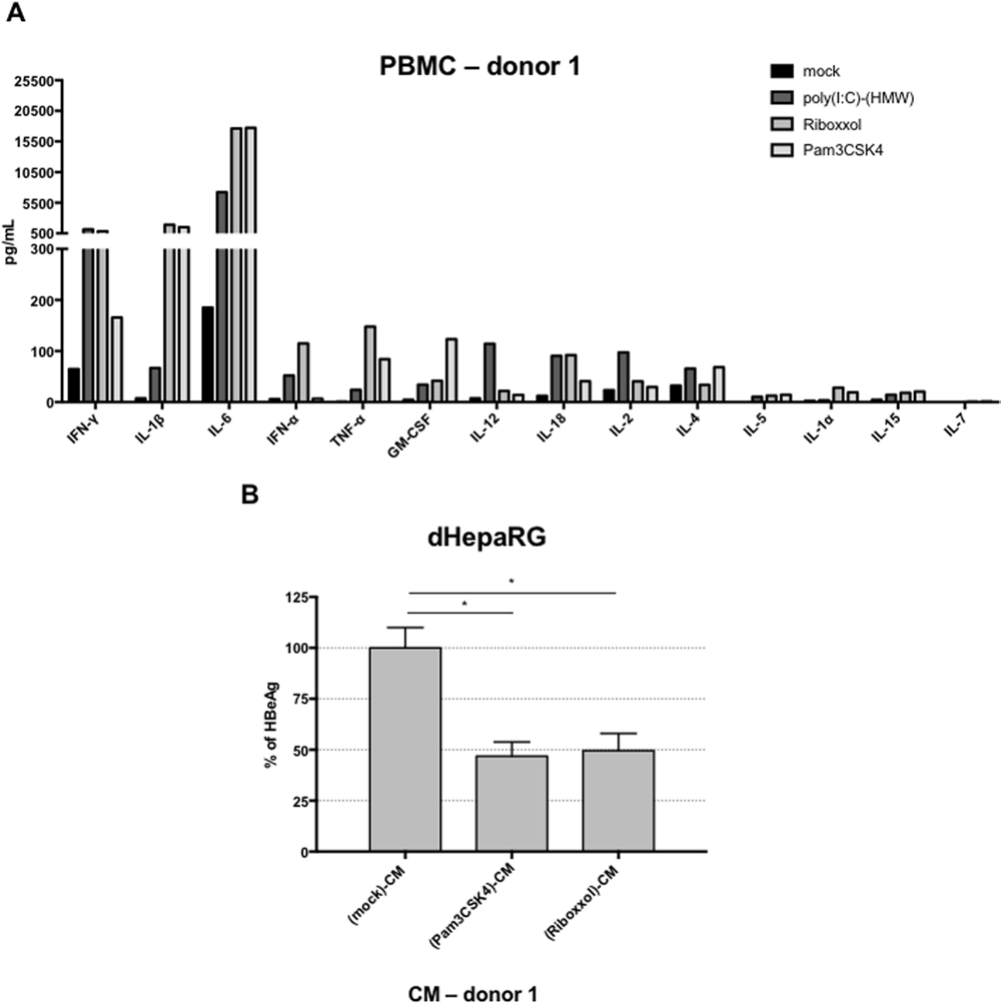
TLR2 and 3 ligands lead to production of inflammatory cytokines by immune blood cells. (**A**) Fresh PBMC from a healthy donor were cultivated and stimulated or not with Pam3CSK4 (5 µg/mL), poly(I:C)-(HMW) (5 µg/mL) or Riboxxol (5 µg/mL) for 24 h. Supernatant were collected and cytokines content was analyzed with Luminex Assay. (**B**) dHepaRG cells were infected by HBV at a multiplicity of infection of 100 viral genome equivalent (vge)/cell for seven days and treated or not during 10 days with the indicated conditioned media (CM) diluted 1/100. Supernatant were collected and HBeAg levels were assessed by ELISA. Results are the mean +/− SD of two independent experiments each performed with two or three biological replicates.

If IL-6 and type-I IFNs production by hepatocytes do not seem to be solely involved in the antiviral effect in our experimental conditions, their production in the liver microenvironment by other cells types are expected to be beneficial for the overall antiviral effect we can anticipate *in vivo*. To this end, we demonstrated that treatment of human peripheral blood mononuclear cells (PBMCs) with Pam3CSK4 strongly induced IL-6, as well as some IL-1β and TNF-α (Fig. 6A). Poly(I:C)-(HMW) induced less IL-6 but more IFN-γ and IFN-α whereas Riboxxol induced all these cytokines, which are known to inhibit HBV^4,15,17–20^ (Fig. 6A). As expected HBeAg secretion by HBV-infected dHepaRG was reduced in the presence of supernatants from these stimulated PBMCs (Fig. 6B). Of note, the amount of cytokines produced by PBMCs and their antiviral effect on HBV-infected hepatocytes may vary from one donor to another (compared Figs 6 to S8). To assess the effect of TLR2-L or TLR3-L *in vivo*, HBV-infected liver-humanized (HuHep) mice mice were intravenously (IV) injected 2 times per week during 3 weeks with Pam3CSK4 or Riboxxol. The dose escalation protocol we have chosen led to very moderate decreases of HBV parameters in this animal model (Fig. S9A) suggesting that either higher doses or a liver-specific delivery of these ligands have to be further tested.

## Discussion

Toll-like receptors are important molecular mediators linking innate and adaptive immunity, and their stimulation by cognate agonists induced an antiviral response in animal models of HBV infection. Indeed, treatments of chimpanzees or woodchucks with TLR7 or TLR9 agonists led to reduction of HBV replication markers^6–8,12^. The strongest antiviral phenotype was obtained with GS-9620, a TLR-7 agonist, in the woodchuck model, with a strong effect of treatment on cccDNA and a long-lasting efficacy associated with an anti-HBsAg seroconversion. These antiviral effects probably resulted from the activation of non-parenchymal cells and subsequent production of anti-HBV cytokines/IFNs, since hepatocytes do not express TLR7 or TLR9 and cannot therefore be directly activated by these agonists^14^. However, hepatocytes express a number of other TLRs and we investigated here if their stimulation with specific agonists could directly inhibit an already established HBV infection in isolated hepatocytes (i.e., in the absence of immune cells).

We demonstrated that ligands of TLR1/2 (Pam3CSK4) and TLR3 (poly(I:C)-(HMW) or Riboxxol activated hepatocytes (PHH or dHepaRG) innate responses and efficiently decreased levels all HBV replication markers, including a strong phenotype on HBV RNAs. This suggests that those treatments are able to prevent transcription of viral RNAs from cccDNA and/or to interfere with their stability. Underlying mechanisms are likely to involve either direct negative epigenetic regulations on cccDNA or a direct IRF3-mediated (i.e., IFN-independent) ISG induction, which would be independent from the production of either IL-6 or IFN-α and their secondary effect on cells via downstream pathways^17,18^, as shown by our neutralization studies. As HBV-infected dHepaRG cells are able to maintain HBV replication for months^21^, we could show that Pam3CSK4 and poly(I:C)-(HMW)-mediated antiviral effect was sustained after arrest of the treatments contrary to the antiviral effect of nucleos(t)ides analogues, thus suggesting a role of epigenetic regulations. In addition, this lack of (or weak) rebound was associated to a slight, but significant, reduction of cccDNA levels measured by both qPCR and Southern blotting in dHepaRG. This suggests that Pam3CSK4 and poly(I:C)-(HMW) could trigger cellular pathways that can subsequently affect cccDNA stability, as shown previously with some cytokines or lymphotoxin-beta agonists^15,20^. It is nevertheless worth noting that the effect on cccDNA of Pam3CSK4, poly(I:C)-(HMW) or Riboxxol could not be confirmed in all PHH donors tested suggesting that (i) either dHepaRG are particularly efficient to trigger innate cellular pathways and/or (ii) the low level of replication of HBV in dHepaRG is more prone to cccDNA degradation and/or (iii) PHH donor heterogeneity (e.g. genetic background, alteration of liver function due the underlying donor disease or treatment) may affect pathways triggering cccDNA destabilization. This also emphasizes the importance of performing experiments with multiple PHH donors. In addition to the direct antiviral effect observed in infected hepatocytes, Pam3CSK4, poly(I:C)-(HMW) and Riboxxol also activated PBMCs to produce different cytokines, which could in turn inhibit HBV replication. Among them, IL-6 might be the main driver of this indirect antiviral effect against HBV since its lower induction in PMBCs from donor 2 stimulated with Riboxxol led to a lower inhibition of HBeAg secretion (Fig. S8B).

Our data are in accordance with experiments performed in HBV transgenic mice or WHV infected woodchuck that identified respectively TLR3 and TLR2 pathways as potential therapeutic targets^22–24^. However, in contrast to agonists of TLR7 (e.g., GS-9620), TLR8 (e.g., GS-9688) and RIG-I/NOD2 (e.g., SB-9200) that can be delivered orally to human, TLR3 and TLR2 ligands cannot and will likely require strategy for delivery since their IV administration with a dose escalation protocol (from 20 to 80 ug during 3 weeks) only led to very moderate decreases of HBV parameters in HBV-infected HuHep mice. In this respect, nanoparticles will be used to reduce the active dose of ligands, protect these ligands from degradation and specifically deliver specifically them to the liver, thereby preventing systemic exposure and potential adverse effects^25^. These ligands may also be used as part of a combination therapies with currently, e.g. with currently used anti-HBV drugs, as well as with immunotherapies, such as therapeutic vaccines and/or checkpoint inhibitors.

## Methods

### Cell culture and HBV infection

HepaRG cells were cultured, differentiated, and infected by HBV as previously described^26^. Primary human hepatocytes (PHH) were freshly prepared from human liver resection obtained from the Centre Léon Bérard (Lyon) with French ministerial authorizations (AC 2013-1871, DC 2013–1870, AFNOR NF 96 900 sept 2011) as previously described^27^. HBV inoculum was prepared from HepAD38^28^ supernatants by polyethylene-glycol-MW-8000 (PEG8000, SIGMA) precipitation (8% final) as previously described^29^. Viral stocks with titer reaching at least 1 × 10^10^ vge/mL were tested endotoxin free. Blood from healthy donors was obtained from “Etablissement Français du Sang”, Lyon France. PBMCs were isolated by gradient centrifugation using Histopaque (Sigma-Aldrich) and cultivated in RPMI medium, 10% FBS, 5% Penicillin-Streptomycin. Poly(I:C)-(HMW), Riboxxol and Pam3CSK4 were added at 5 µg/mL concentration. PBMCs were incubated for 24 h at 37 °C and 5%CO2 after TLRs stimulation.

### Reagents

The following PRR agonists were purchased from InvivoGen and used at the indicated final concentration unless stated otherwise in the figure legends: Pam3CSK4 (TLR-1/2 agonist, 0.5 μg/mL), Poly(I:C)-HMW (TLR3-agonist, 5 μg/mL), LPS (TLR-4 agonist, 0.5 μg/mL), FLA-BS (TLR-5 agonist, 1 μg/mL), FSL-1 (TLR-2/6-agonist, 1 μg/mL), Imiquimod (R837, TLR-7 agonist, 5 μg/mL), CL264 (TLR-7 agonist 5 μg/mL), ssRNA40/lyoVecTM (ssRNA, TLR-8 agonist, 1 μg/mL), 2395 (CpG ODN-2395 (class-C), TLR-9 agonist, 5 μM). Riboxxol, recombinant IL6 and rhPEG-IFN-α (Roferon) were purchased respectively from Riboxx life science, R&D Systems and Roche. Neutralizing anti-IFNRA1 antibodies (IgG2a, #21385-1) and their isotype control antibodies (IgG2a, #281-010) were purchased respectively from PBL assay Science and Ancell. Neutralizing anti-IL-6 antibodies (IgG1, #501110) and their isotype control antibodies (IgG2, #400414) were purchased from Biolegend. Transfection of siRNA (SMARTpoolTM from Dharmacon/GE) were performed using Dharmafect-1 transfection reagent as recommended by the manufacturer (Darmacon/GE). siRNA against TLR3(# L-007745-00) RIG-I (# L-012511-00), MDA5 (# L-013041-00) and HCV (as siRNA control) was used at final concentration of 25 nM.

### Western blot analysis

Cells were harvested in RIPA lysis buffer (NaCl 150 mM, Tris HCl pH = 8,0 50 mM, SDS 0,1%, NP40 1%, Na Deoxycholate 0,5%) containing protease inhibitors (Protein Cocktail Inhibitors from Sigma-Aldrich, NaF 10 mM, Na Orthovanadate 10 mM). Clarified lysates were subjected to 10% SDS-PAGE and Western Blot transfer onto PVDF or nitrocellulose membranes using the TransTurbo Blot apparatus according to the manufacturer (Biorad). Primary antibodies are the TLR3 antibody (D10F10, #6961) RIG-I antibody (D14G6, #3743) MDA5 antibody (D74E4, #5321) from Cell Signaling and the anti-huActin (clone C4, #08691002) from MP Biomedicals. Secondary HRP antibodies anti-rabbit and anti-mouse were purchased from Sigma-Aldrich.

### Nucleic acid extractions, reverse transcription and qPCR analyses

Total RNA and total DNA were respectively extracted from cells with the NucleoSpin RNA II kit and Nucleospin® 96 tissue kit according to the manufacturer’s instructions (Macherey-Nagel). RNA reverse transcription was performed using the Superscript III RT (Life Technologies). Quantitative PCR for HBV were performed using HBV specific primers and normalized to PRNP housekeeping gene as previously described^15^.

### cccDNA detection

After total DNA isolation and T5 exonuclease digestion (NEB, Fance) digestion for 1 h, cccDNA was quantified as previously described^30^. Alternatively, cccDNA was analyzed by Southern blot after Hirt extraction as described previously^31^.

### Enzyme-linked Immunoassay for IL-6, IP10, HBeAg, HBsAg, and ApoB detection in cell supernatant

Human IL-6 and human IP-10 cytokines were detected in the supernatants using the DuoSet® ELISA kit according to the manufacturer (R&D Systems). HBeAg and HBsAg were detected in the supernatant of HBV-infected cells using the Autobio kit according to the manufacturer (AutoBio, China). ApoB secretion was detected in the supernatant of cells using the Total Human Apolipoprotein B assay according to the manufacturer (AlerCHEK, USA).

### Measurement of cytokines by Luminex

IL-12p70, GM-CSF, IFN-α, IFN-γ, IL-1α, IL-1β, IL-1RA, IL-13, IL-15, IL-18, IL-2, IL-31, IL-4, IL-5, IL-6, IL-7, TNF-α and TNF-β were measured with a ProcartaPlex Human Th1/Th2 & Cytokine Panel 1 C (18 plex) kit (eBiosciences). Samples were analyzed using a Bio-Plex® 200 System (Bio-Rad, Hercules, CA) according to the manufacturer’s instructions.

### Viability/cytotoxicity assays

Neutral red uptake assay and Sulforhodamine staining to estimate cell viability/cytotoxicity were performed as previously described^4^.

### *In vivo* experiments

All experiments were performed in accordance with the European Union guidelines for approval of the protocols by the local ethics committee (Authorization Agreement C2EA-15, “Comité Rhône-Alpes d’Ethique pour l’Expérimentation Animale”, Lyon, France - APAFIS#1570-2015073112163780). Primary Human Hepatocytes (PHH, Corning, BD Gentest) were injected in FRG mice intrasplenically 48 h after adeno-uPA conditioning^32^. Mice were subjected to NTBC cycling during the liver repopulation process. Mice with HSA levels >19 mg/ml, as determined using a Cobas C501 analyzer, Roche Applied Science, were infected by IP injection with 5 × 10^8^ vge/mL of HBV. Six weeks later, mice were injected intravenously twice a week during 3 weeks with Pam3CSK4 or Riboxxol (20 ug per mouse per injection the first week, 40 ug per mouse per injection the second week and 80 ug per mouse per injection the third week). Sera were collected at different time before and after treatments. Mice were sacrificed 10,5 weeks post HBV infection and liver pieces were flash frozen in liquid nitrogen and kept at −80 °C before further processing. HBV viremia was determined by extraction of DNA from sera (supplemented with a known amount of non-HBV DNA; pUC19 plasmid from New England Biolabs) using the NucleoSpin® RNA Virus kit (Macherey Nagel) followed by qPCR. For each serum, quantity of viral genome equivalent was calculated according to a standard curve (build with dilutions of a plasmid containing the HBV sequence) and normalized to the ratio between the actual and theoretical amount of recovered pUC19 DNA. Total DNA were extracted from liver pieces and cccDNA amount was quantified after T5 digestions as described previously^33^.

### Statistical analysis

Statistical analyses were performed using the XLStat software and Kruskal-Wallis tests with multiple comparison respect to non-treated cells (Dunn’s post-test). For all tests, a p value ≤ 0,05 was considered as significant. *Correspond to p value ≤ 0.05; **correspond to p value ≤ 0.01; ***correspond to p value ≤ 0.001.

## Electronic supplementary material

Supplementary figures
