# Supplementary material for: Direct antiviral properties of TLR ligands against HBV replication in immune-competent hepatocytes
Source: Sci Rep. 2018 Mar 29;8:5390. doi: 10.1038/s41598-018-23525-w (PMC5876392; doi:10.1038/s41598-018-23525-w)
Supplement: Supplementary file 1 — Supplementary figures [file 41598_2018_23525_MOESM1_ESM.pdf]

Supplementary figures

Direct antiviral properties of TLR ligands against HBV replication in immune-competent hepatocytes

Julie Lucifora[1,*,$], Marc Bonnin[1,$], Ludovic Aillot[1], Floriane Fusil[2], Sarah Maadadi[1], Laura Dimier[1], Maud Michelet[1], Océane Floriot[1], Anaïs Ollivier[2], Michel Rivoire[3], Malika Ait-Goughoulte[4], Stéphane Daffis[5], Simon P. Fletcher[5], Anna Salvetti[1], François-Loïc Cosset[2], Fabien Zoulim[1,6] and David Durantel[1,*]

[1] INSERM, U1052, Cancer Research Center of Lyon (CRCL), Université de Lyon (UCBL1), CNRS UMR_5286, Centre Léon Bérard, Lyon, France
[2] CIRI – International Center for Infectiology Research, Inserm, U1111, Université Claude Bernard Lyon 1, CNRS, UMR5308, Ecole Normale Supérieure de Lyon, Univ Lyon, F-69007, Lyon, France
[3] INSERM U1032, Centre Léon Bérard (CLB), Lyon, France
[4] Roche Pharma Research and Early Development (pRED), Roche Innovation Center Basel, F. Hoffmann-La Roche, 4070 Basel, Switzerland
[5] Gilead Sciences, Inc., Foster City, CA, USA;
[6] Department of Hepatology, Croix-Rousse Hospital, Hospices Civils de Lyon, Lyon, France

[$] equal contribution

***Correspondence:**
Dr. David Durantel and Dr. Julie Lucifora
Centre de Recherche en Cancérologie de Lyon (CRCL), UMR Inserm 1052 - CNRS 5286, 151 cours Albert Thomas, 69424 Lyon Cedex 03, France ; Phone: + 33 4 72 68 19 70 ; Fax : +33 4 72 68 19 71 ; E-mail: david.durantel@inserm.fr and julie.lucifora@inserm.fr

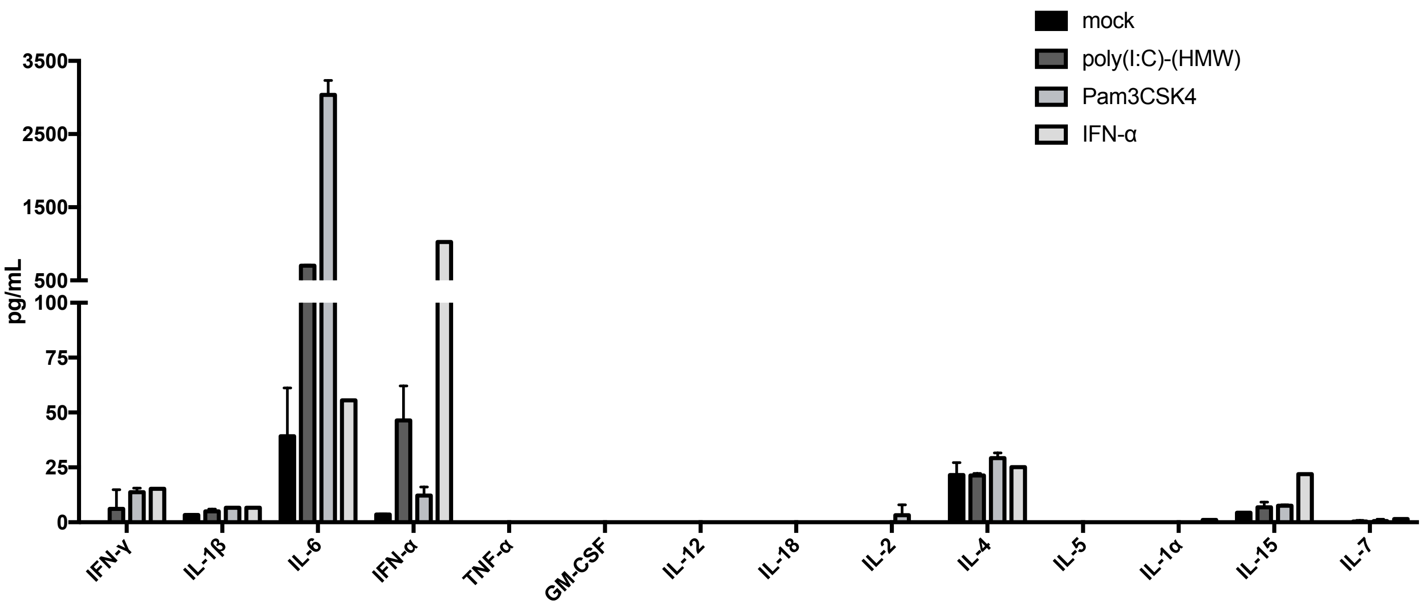

Figure S1: **Cytokines secretion by dHepaRG stimulated with poly(I:C)-(HMW) or Pam3CSK4.** dHepaRG cells were infected by HBV at a multiplicity of infection of 100 viral genome equivalent (vge)/cell for seven days, treated or not with Pam3CSK4 (0,5 ug/mL), poly(I:C)-(HMW) (5 ug/mL) or PEG-IFN-α (1000 IU/mL) for 24h. Supernatants were collected and amount of the indicated cytokines were measured by Luminex assay. Results are the mean +/- SD one experiment performed with two biological replicate.

dHepaRG

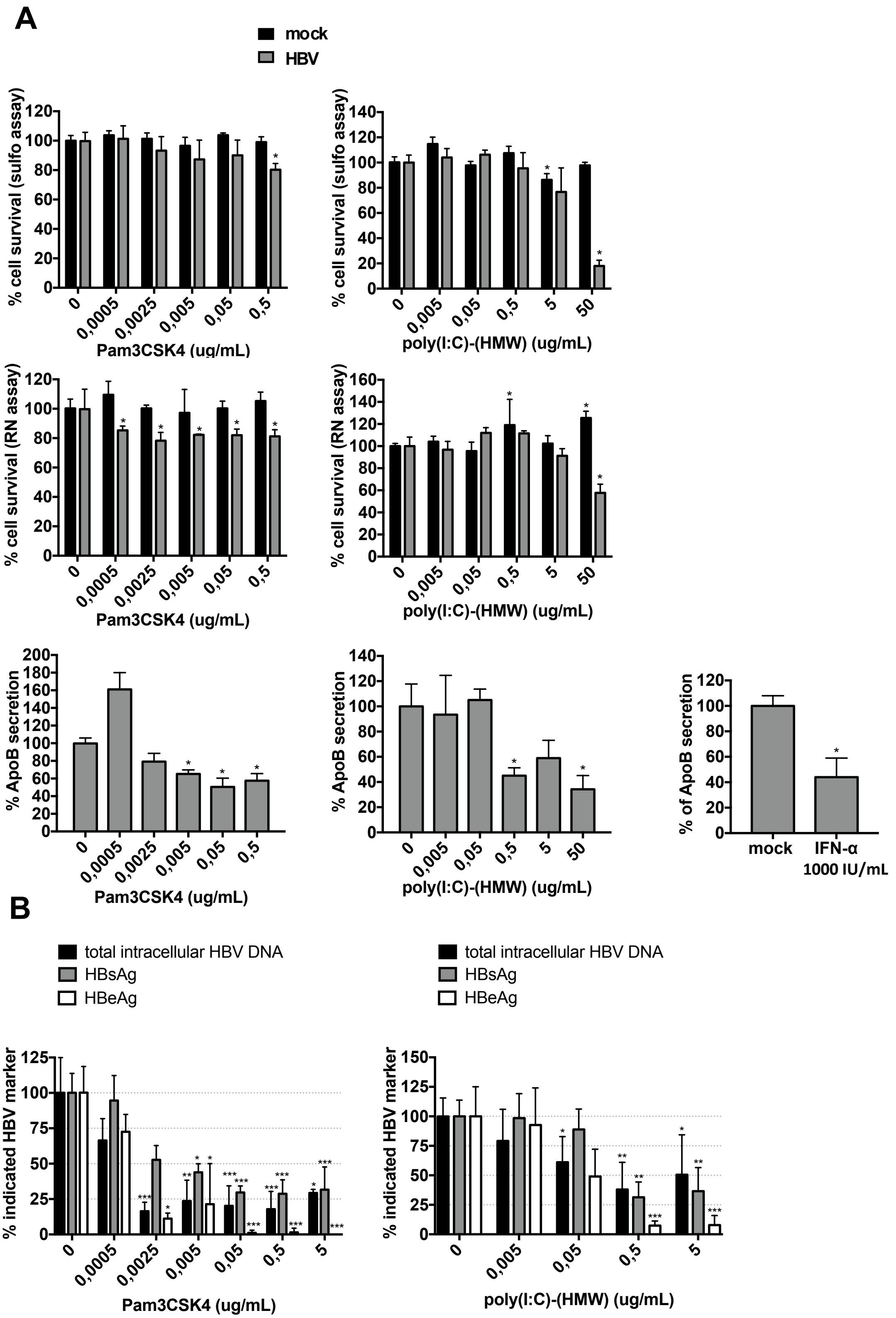

Figure S2: **Pam3CSK4 and poly(I:C)-(HMW) inhibit HBV in HBV-infected dHepaRG in a dose dependent manner without toxicity**. dHepaRG were infected by HBV at a multiplicity of infection of 100 viral genome equivalent (vge)/cell for seven days and treated with the indicated molecules at the indicated concentrations for seven more days (2 treatments). At the end of the experiments, (A) cells survival rates was assessed by sulforhodamin or neutral red assays, (B) total DNAs were extracted and total intracellular HBV DNA amounts were evaluated by qPCR analyses. Supernatant were collected and (A) ApoB, (B) HBeAg or HBsAg levels were assessed by ELISA. Results are the mean +/- standard deviation (SD) of three independent experiments each performed with three biological replicate.

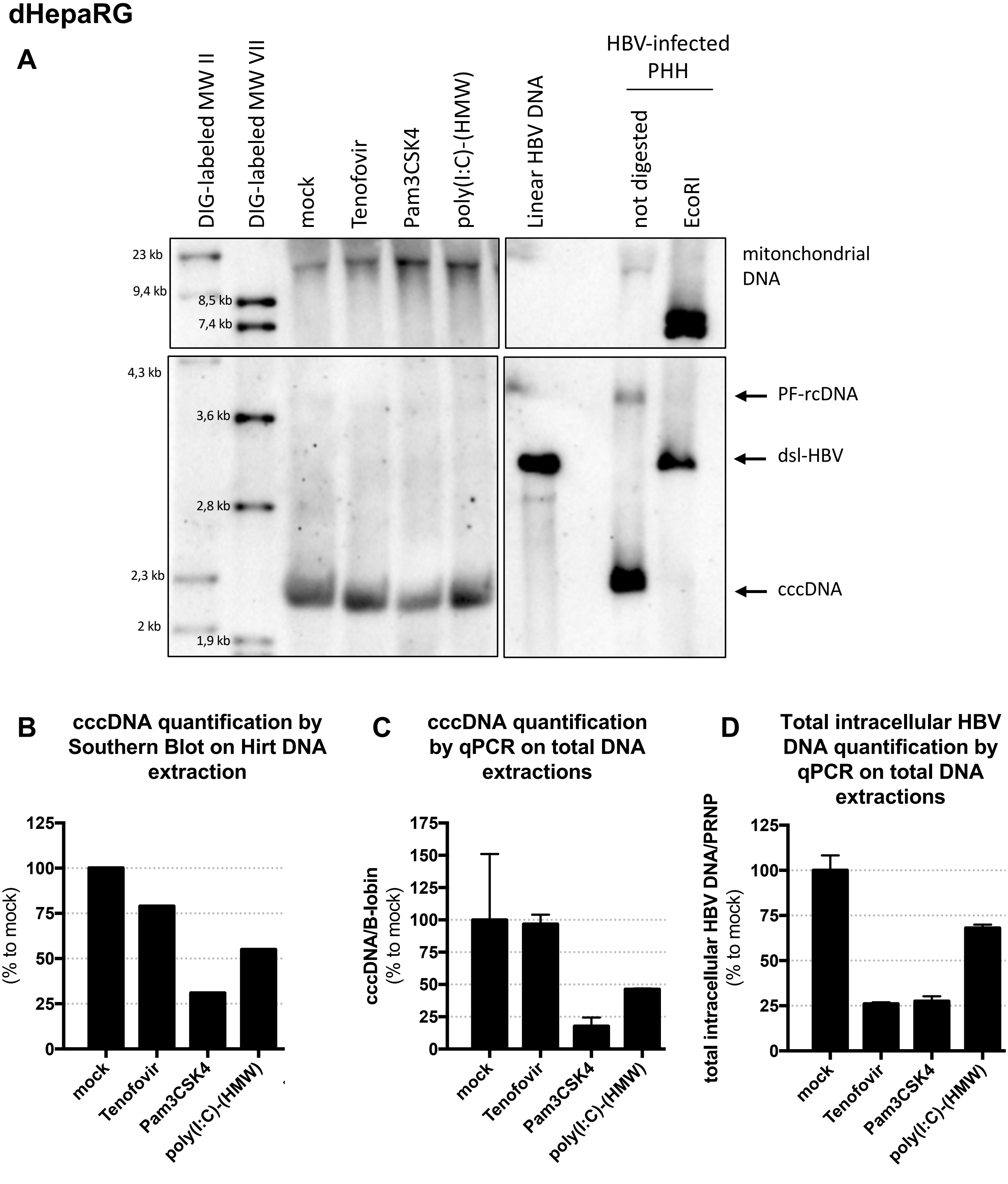

Figure S3: **Pam3CSK4 and poly(I:C)-(HMW) treatments decreased cccDNA levels in HBV-infected dHepaRG**. dHepaRG were infected by HBV at a multiplicity of infection of 100 viral genome equivalent (vge)/cell for seven days and treated with Tenofovir (10 uM), Pam3CSK4 (0,5 ug/mL) or poly(I:C)-(HMW) (0,5 ug/mL) for seven more days (2 treatments). At the end of the experiments, cells were harvested, (A, B) DNAs were extracted following a Hirt procedure or (C, D) a total DNA extraction procedure and submitted to (A) Southern blot analyses using HBV-DIG labeled probes or (C, D) qPCR analyses to detect (C) cccDNA or (D) total intracellular HBV DNAs. (B) cccDNA and mitochondrial DNA were quantified on the southern blot analyses using the « ImageLab » software from Bio-Rad.

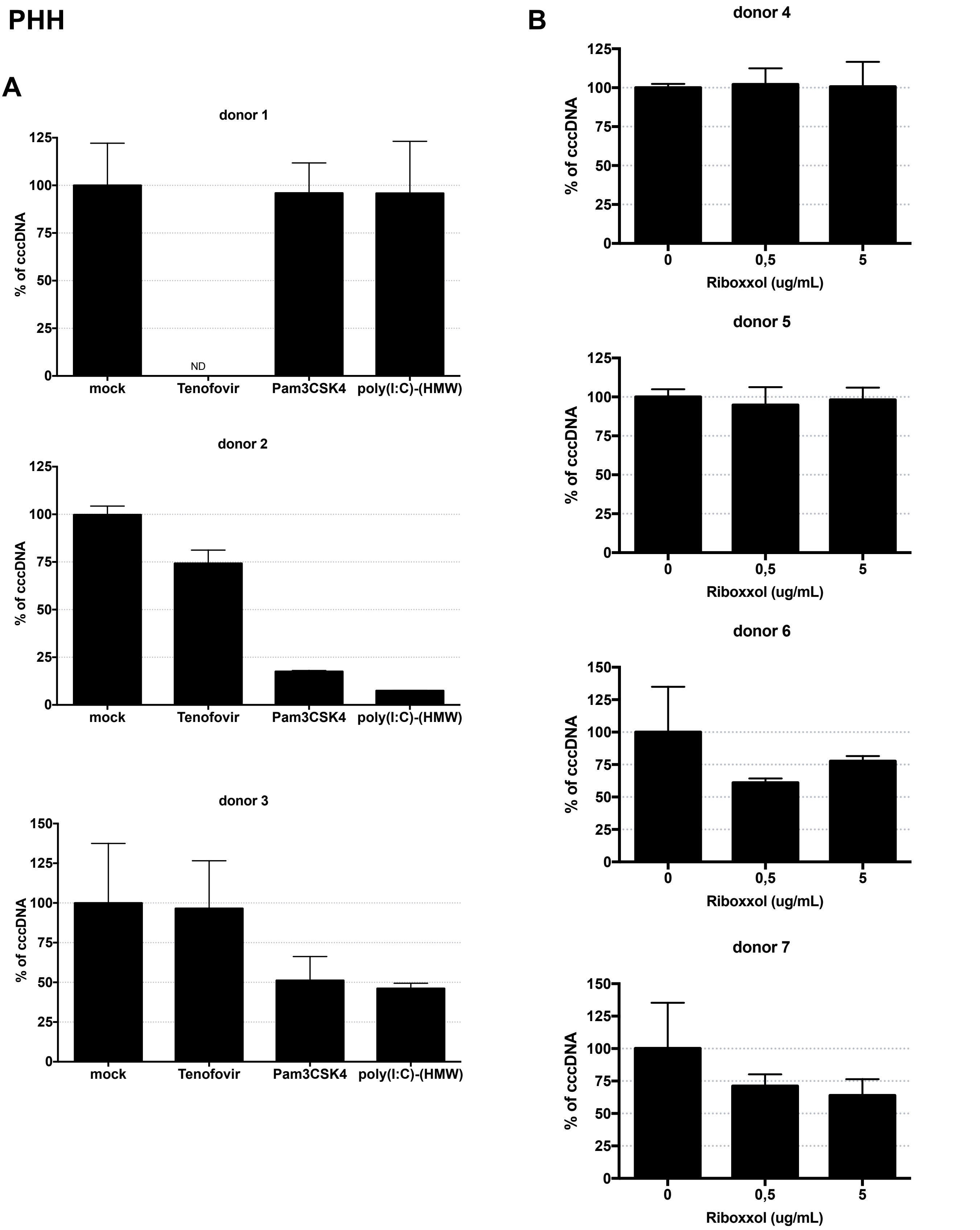

Figure S4: **Effect of Pam3CSK4, poly(I:C)-(HMW) and riboxxol treatments on cccDNA levels in HBV-infected PHH**. PHH were infected by HBV at a multiplicity of infection of 100 viral genome equivalent (vge)/cell for four days and treated with Tenofovir (10 uM), Pam3CSK4 (0,5 ug/mL), poly(I:C)-(HMW) (0,5 ug/mL) or Riboxxol (indicated concentrations) for seven more days (2 treatments). At the end of the experiments, cells were harvest, total DNA**s** were extracted and cccDNA amounts were evaluated by qPCR analyses. For each donor, results are the mean +/- SD one experiment performed with three biological replicate.

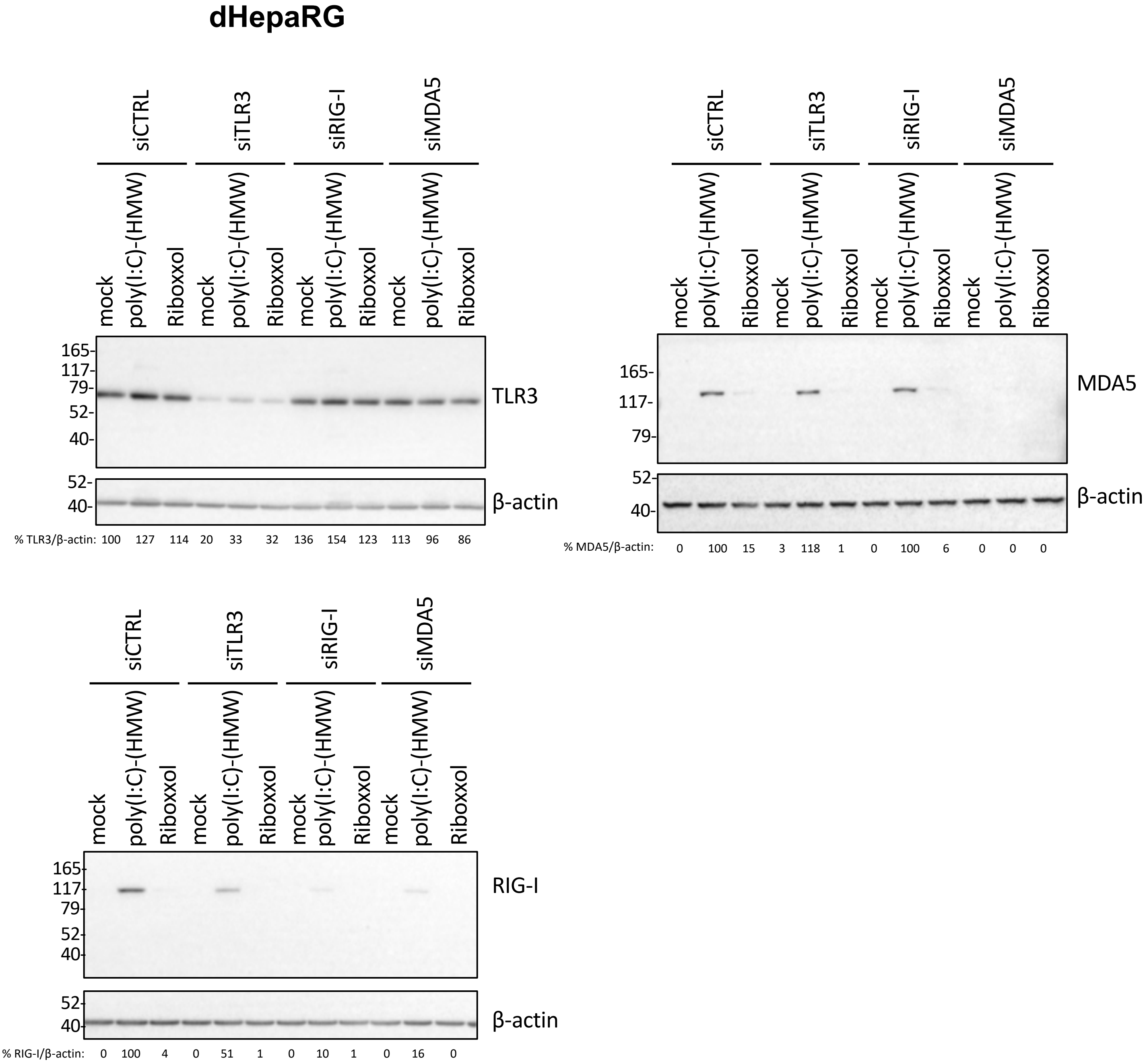

Figure S5: **TLR3, MDA5 and RIG-I protein levels after siRNA transfection.** Uninfected dHepaRG cells were transfected with the indicated siRNA. 14 days later, cells were stimulated with 10 µg/ml of the indicated ligands. Cells were lyzed 24h later and TLR3, MDA5 or RIG-I protein levels were analyzed by western blot. Blots presented here are representative of three independent experiments.

dHepaRG

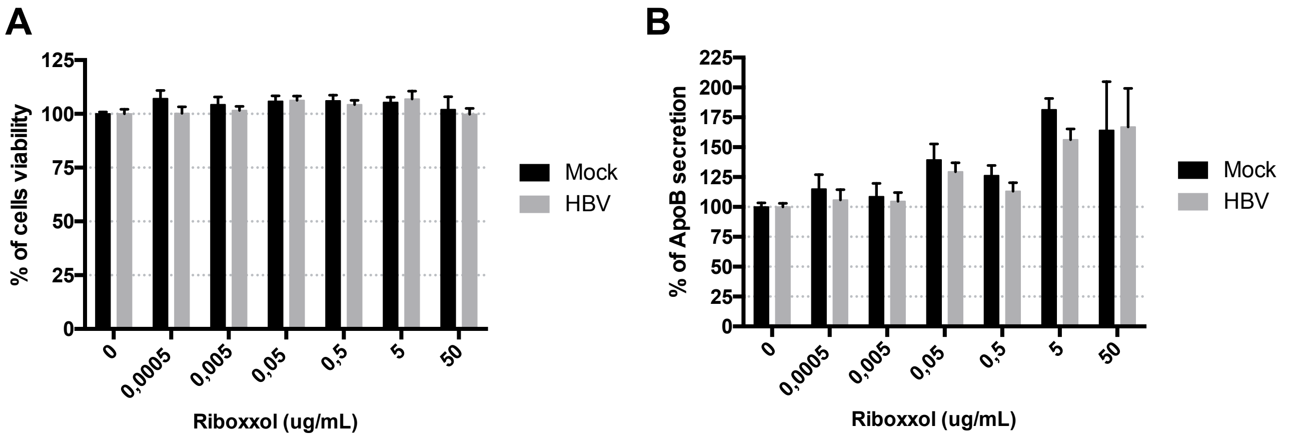

Figure S6: **Treatments with Riboxxol did not impact on dHepaRG survival and differentiation status.** dHepaRG were infected by HBV at a multiplicity of infection of 100 viral genome equivalent (vge)/cell for seven days and treated with the riboxxol at the indicated concentrations for seven more days (2 treatments). At the end of the experiments, (A) cells survival rates was assessed by sulforhodamin assay, (B) supernatant were collected and ApoB levels were assessed by ELISA. Results are the mean +/- standard deviation (SD) of three independent experiments each performed with three biological replicate.

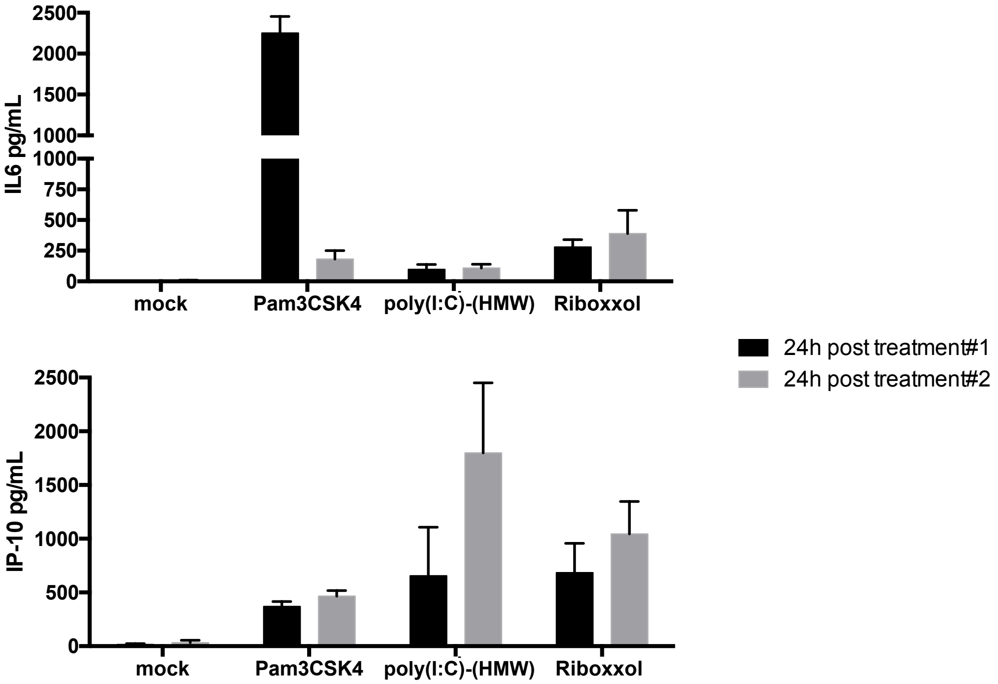

Figure S7: **Riboxxol induced secretion of both IL-6 and IP-10.** dHepaRG cells were infected by HBV at a multiplicity of infection of 100 viral genome equivalent (vge)/cell for seven days, treated or not with Pam3CSK4 (0,5 ug/mL), poly(I:C)-(HMW) (5 ug/mL) or Riboxxol (50 ug/mL) for seven days (2 treatments). Supernatants were collected and amount of IL6 and IP-10 were quantified by ELISA. Results are the mean +/- SD of three independent experiments each performed with three biological replicate.

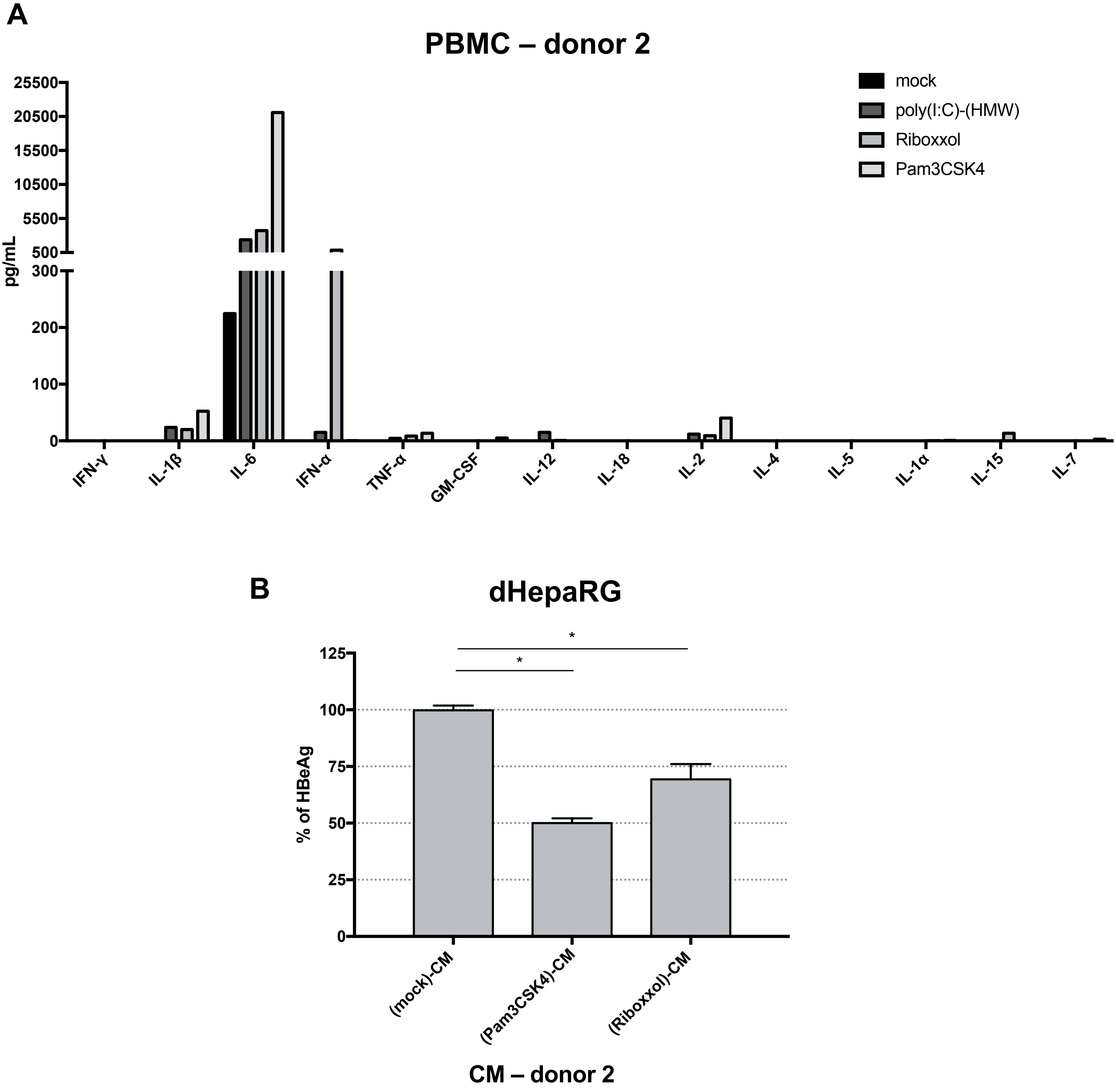

Figure S8: **TLR2 and 3 ligands lead to production of inflammatory cytokines by immune blood cells**. (A) Fresh PBMC from a healthy donor were cultivated and stimulated or not with Pam3CSK4 (5 µg/mL), poly(I:C)-(HMW) (5 µg/mL) or Riboxxol (5 µg/mL) for 24h. Supernatant were collected and cytokines content was analyzed with Luminex Assay. (B) dHepaRG cells were infected by HBV at a multiplicity of infection of 100 viral genome equivalent (vge)/cell for seven days and treated or not during 10 days with the indicated conditioned media (CM) diluted 1/100. Supernatant were collected and HBeAg levels were assessed by ELISA.

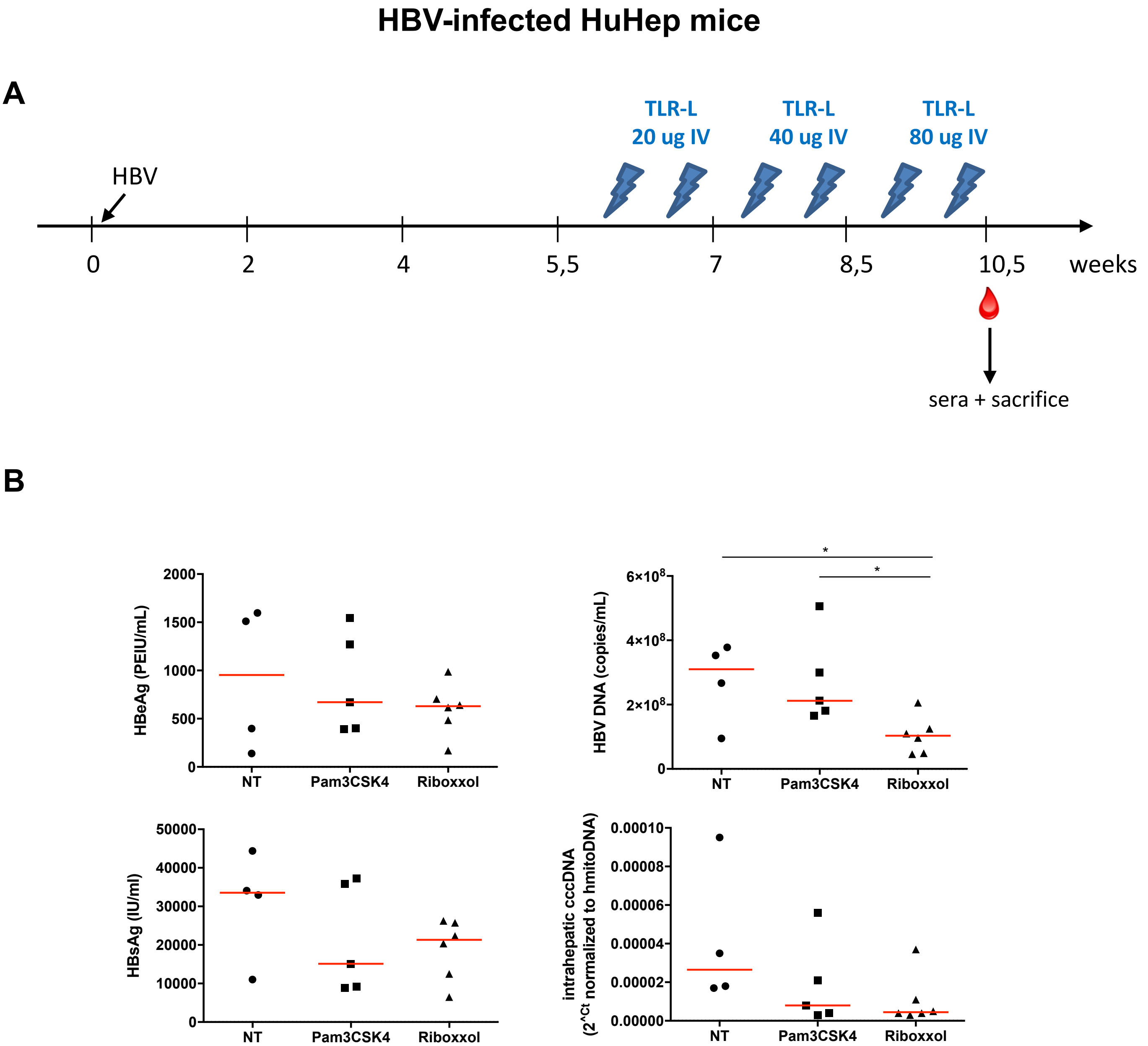

Figure S9: **Effect of Pam3CS4K and Riboxxol in HBV-infected HuHep mice.** HuHep mice were infected IP with HBV and treated IV with TLR-L (Pam3CSK4 or Riboxxol) or not (NT; no treatment) (A) according to the scheme. Mice were sacrificed after 3 weeks of treatment and (B) levels of HBV markers (HBeAg, HBSAg, viremia and intrahepatic HBV cccDNA) were assessed by ELISA, qPCR or RT-qPCR. Each dot represents one animal and red lines the median from the 4-5 animals.

A

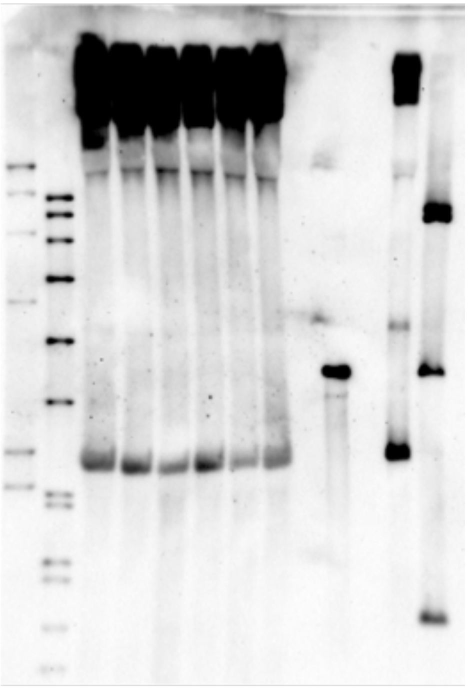

B

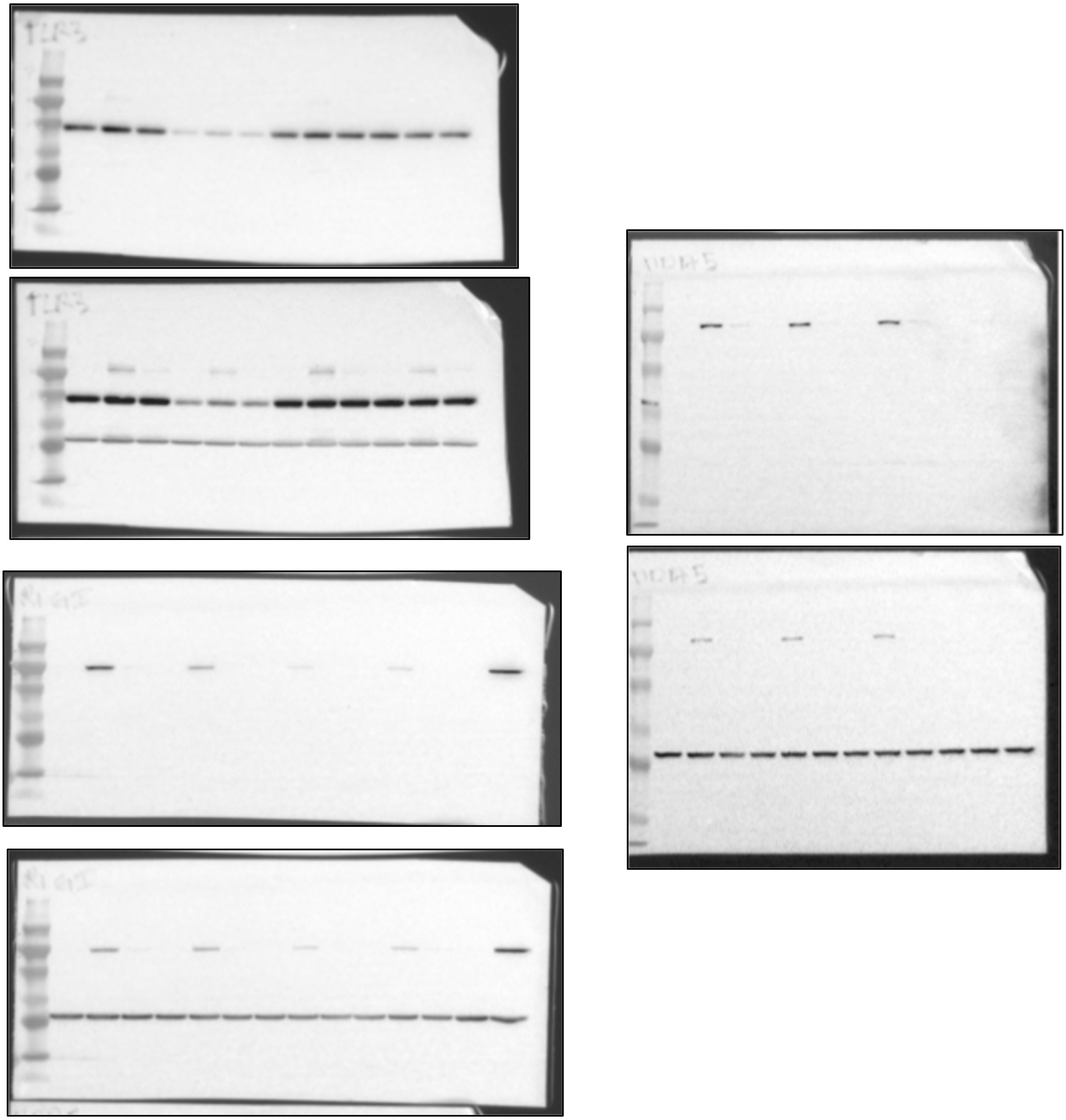

Figure S11: **Full blots** from (A) fig S3A, (B) fig S5
